# Hospital Hygiene Paradox: MRSA and Enterobacteriaceae Colonization Among Cleaning Staff in a Tertiary Hospital in Saudi Arabia

**DOI:** 10.3390/medicina61030384

**Published:** 2025-02-22

**Authors:** Maher S. Alqurashi, Asma A. Sawan, Mahmoud M. Berekaa, Basavaraja C. Hunasemarada, Mohammed D. Al Shubbar, Abdulaziz A. Al Qunais, Ammar S. Huldar, Loay M. Bojabara, Saud A. Alamro, Ayman A. El-Badry

**Affiliations:** 1 Department of Microbiology, College of Medicine, Imam Abdulrahman Bin Faisal University, Dammam 31441, Saudi Arabia; msalqurashi@iau.edu.sa (M.S.A.); aasawan@iau.edu.sa (A.A.S.); mberekaa@iau.edu.sa (M.M.B.); nimmibasu@uod.edu.sa (B.C.H.); aaelbadry@iau.edu.sa (A.A.E.-B.); 2 College of Medicine, Imam Abdulrahman Bin Faisal University, Dammam 31441, Saudi Arabia; mdshubbar0@gmail.com (M.D.A.S.); abdulazizalqunais@gmail.com (A.A.A.Q.); loaybojbara5@gmail.com (L.M.B.); saudalamr@hotmail.com (S.A.A.)

**Keywords:** hospital cleaning staff, hospital hygiene, methicillin-resistant *Staphylococcus aureus* (MRSA), Enterobacteriaceae, hospital-acquired infections (HAIs), nosocomial infections

## Abstract

*Background and Objectives:* Despite extensive research on the sources and transmission pathways of Hospital-Acquired Infections (HAIs), the role of cleaning staff as potential vectors has been under-explored. This study addresses the gap by examining the cleaning staff’s role in nosocomial infection transmission, focusing on pathogenic bacteria and fungi colonization. Identifying potential pathogens harbored by cleaning staff that carry the risk of causing HAIs, especially methicillin-resistant *Staphylococcus aureus* (MRSA) and Enterobacteriaceae. *Materials and Methods:* This current cross-sectional study was conducted on 96 cleaning staff at King Fahad Hospital of the University and Family and Community Medicine-Centre, Eastern Province, Saudi Arabia. Sample collection targeted hands and nostrils using cotton swabs, followed by laboratory testing, including MALDI-TOF mass spectrometry for bacterial identification, and the cefoxitin disc diffusion test for the screening of MRSA. *Results:* In total, the occurrence of MRSA colonization was 9.4% while Enterobacteriaceae colonization was 15.6%. No significant correlation was found for MRSA among departments or between day and night shifts. Alternatively, individuals working in the ICU and the operating room showed higher chances of being colonized by Enterobacteriaceae, and a notable connection was identified between Enterobacteriaceae and day shifts. Ultimately, the frequency of handwashing reduced the chances of being colonized by MRSA and Enterobacteriaceae, underscoring the significance of good hygiene practices. *Conclusions:* The study highlights the potential role of cleaning staff in transmitting HAIs, indicating a need for further research and consideration of enhanced hygiene protocols in hospital settings.

## 1. Introduction

Hospital-acquired infections (HAIs) are infections that patients acquire while receiving treatment in a healthcare facility. These infections are typically not present during the time of admission, and usually develop at least 48 h after admission [1]. According to the World Health Organization (WHO), HAIs represent a significant challenge to healthcare systems worldwide, affecting both developed and developing countries and contributing to considerable morbidity and mortality. Epidemiological data indicate that the prevalence of HAIs varies across regions. In the Americas, approximately 3.2% of hospitalized patients develop an HAI. This prevalence increases in the European Union, ranging from 6.5% to 8.9%, while the highest frequency is reported in the Eastern Mediterranean region, where 11.2% of patients acquire an HAI. Furthermore, the WHO global report highlights that among patients who develop HAI-associated sepsis, the mortality rate is 24.4%, with a substantial increase to 52.3% among those receiving treatment in intensive care units (ICUs) [2]. Furthermore, these infections are not only associated with high mortality and morbidity but also have some significant economic implications, as some studies show that HAIs are linked to high economic burdens [3,4]. Taking the United States (US) as an example, the overall direct cost of HAIs is estimated to range from 28 to 45 billion US dollars annually [5].

Numerous bacterial and fungal pathogens have been identified as causes of HAIs. Common pathogens include Clostridioides difficile, Staphylococcus aureus, Klebsiella pneumoniae, Klebsiella oxytoca, Escherichia coli, and Pseudomonas aeruginosa [6,7]. These pathogens are often characterized by a higher rate of antimicrobial resistance, which complicates treatment and increases the risk of severe outcomes particularly, are Methicillin-resistant Staphylococcus aureus (MRSA), Carbapenems-resistant Enterobacteriaceae (CRE), and Vancomycin-resistant Enterococcus (VRE). Antimicrobial resistance makes HAIs difficult to treat and is associated with high morbidity and mortality rates [8,9,10,11]. Infections like MRSA and Carbapenem-resistant Klebsiella are associated with high levels of mortality, and are of special interest when discussing HAIs [12,13]. Additionally, fungal infections, particularly those caused by Candida species, are a significant cause of HAIs, especially in immunocompromised patients. Although the most common type is Candida albicans, it is less likely to possess antifungal resistance. However, other types of Candida, like Candida auris, have been reported to possess multidrug resistance [14,15].

Transmission pathways for HAIs are varied and can include inanimate objects such as patient surroundings and surfaces or animate sources, predominantly healthcare staff [16]. Healthcare staff have traditionally been considered the main vectors of pathogen transmission in medical settings. Research across various studies involving healthcare workers has provided substantial scientific evidence that pathogenic bacteria can colonize different parts of their bodies and their clothing, highlighting the significant importance of personal hygiene [17,18,19,20].

While infection control training and education are commonly directed towards healthcare staff, the WHO also recommends extending these efforts to all staff working in healthcare facilities, including cleaning and housekeeping personnel [2]. Despite this recommendation by the WHO, there remains a persistent underestimation of the role of cleaning staff in the transmission of HAIs. This neglect is evident from the paucity of research dedicated to this group of potential carriers. By investigating the involvement of cleaning staff in the spread of HAIs, this research aims to address a critical gap in the literature on infection control and prevention. This research would lead to an enhancement of hospital hygiene practices, improve infection control policy, and improve patient outcomes in the face of a global challenge posed by anti-microbial-resistant pathogens.

## 2. Materials and Methods

### 2.1. Study Design and Population

This research applied a cross-sectional study on prevalence of MRSA and Enterobacteriaceae colonization among cleaning Staff at a tertiary hospital and its affiliated family medicine center in Al-Khobar, Saudi Arabia. The study encompassed cleaning personnel who have potential contact with patients, including those stationed in various wards such as surgical, medical, pediatrics, obstetrics and gynecology, the emergency department, intensive care units (ICU; medical, surgical, and cardiac), psychiatry wards, operating rooms, outpatient clinics, and the family medicine center. Also, the study covered all cleaning staff across different shifts, both day and night. However, cleaning staff working in administrative buildings were not included in the study.

Sampling was conducted between 14 January 2024 and 4 March 2024 and was planned to conclude with the whole hospital staff which comprises 113 members. However, a total sample size of 96 participants was reached. The rest either refused participation or could not be reached. Before sampling, informed consent was obtained from each participant, alongside a questionnaire to collect information regarding their demographic characteristics, which included age, gender, and nationality. Moreover, other work-related data which included the type of shift, the work location, frequency of handwashing, and method of handwashing (alcohol vs. soap and water) were also collected.

### 2.2. Sample Collection and Transportation

In the current study, hands and nostrils were used as the major sites for isolation of the probable healthcare-associated contaminants [21]. Each worker provided two samples namely: one from hand and another from nose using sterile cotton swabs moistened with normal saline. For nasal samples, a single swab was used to collect specimens from both anterior nares. For hand sampling, a single swab systematically covered the palms, around the nails, and the spaces between the fingers of both hands. Samples were taken during the working hours for morning and evening shifts. Subsequently, each swab was then securely placed in a sterile tube, stored in a cold box at 4 °C, and transported to the laboratory within 1–2 h of collection for further processing.

### 2.3. Laboratory Analysis

For selective isolation of the probable healthcare-associated pathogens, each sample was inoculated in MacConkey agar, Blood agar, (Saudi Prepared Media Laboratory Riyadh, Saudi Arabia) and CHROMagar™ *Candida* (CHROMagar, Saint-Denis, France). Then incubated under aerobic conditions for 48 h. Further purification was then carried out as needed. Each colony was subcultured on identical selective chromogenic agar medium. For further identification of each bacterial isolate, the VITEK ^®^ MS VITEK MS PRIME (bioMérieux, Marcy l’Etoile, France) was employed according to the manufacturer’s instructions. This machine is an automated mass spectrometry microbial identification system, based on matrix-assisted laser desorption ionization-time of flight (MALDI-TOF) technology [21]. For identification, each colony of the isolated bacterial candidate was applied on a target plate, then mixed with matrix solution and inserted into the machine. After processing, each bacterial candidate was automatically identified according to the pattern of matching between mass spectrum of the unknown and the registered bacteria in database, based on the score of the confidence level. This method can identify each bacterial candidate from the genus and up-to-the-species level or probably remain unidentified if confidence level is low.

Also, identification of *Candida* species was done by CHROMagar™ *Candida*, which yields specifically colored colonies for each species [22]. On the other hand, this study’s antimicrobial resistance scope was explicitly focused on the identification of *S. aureus* isolates. Screening for MRSA was chosen specifically since it is highly prevalent [23] and is the most commonly recorded antibiotic-resistant organism in many hospitals [24]. For screening of MRSA distribution among the isolated *S. aureus* contaminants, cefoxitin disc diffusion test was performed.

### 2.4. Statistical Analysis

Generally, data collected were exposed to statistical analysis using IBM SPSS version 27.0.1. Simple descriptive statistics of the sociodemographic characteristics and other categorical variables (including the prevalence of the organisms), in the form of frequencies and percentages were calculated and tabulated. Furthermore, inferential statistical analysis was also carried out; this involved the use of Fischer’s Exact to assess the association between the colonization prevalence of MRSA, and Enterobacteriaceae with certain sociodemographic characteristics. In addition, binary logistic regression models were created to find out the predictors of colonization of MRSA, methicillin-sensitive *Staphylococcus aureus* (MSSA), and Enterobacteriaceae. Significance was established at a *p* value of 0.05 or less indicating a 95% confidence interval.

## 3. Results

### 3.1. Sociodemographic Characteristics

The study involved a total of 96 cleaning staff from King Fahad University Hospital and Family and Community Medicine Centre, Eastern Province, Saudi Arabia. The mean age of participants was 42.2 years, with a standard deviation of 8.7. Age distribution varied, with the majority falling within the 41–50 age range (41.7%). Gender distribution skewed towards males, constituting 77.1% of the sample. Most participants were of Bangladeshi nationality (41.7%). Regarding work shifts, 62.5% worked during the day. In terms of departmental distribution, most participants worked in inpatient settings (75.0%), including various wards, emergency rooms, and ICUs. Outpatient settings accounted for 25.0% of participants, including outpatient clinics and family medicine centers. Handwashing practices varied, with the majority washing hands after specific tasks (69.8%) using soap and water (85.4%) (Table 1).

### 3.2. Distribution of Bacterial Contaminants in Cleaning Staff

Overall, a total of 283 bacterial isolates were isolated from both nasal and hand samples. A total of 134 isolates were obtained from nasal samples while approximately 53% of contaminants (comprising 149 isolates) were collected from hand samples.

### 3.3. Colonization Prevalence of Bacterial Contaminants in Nose and Hands of Participants

Table 2a presents the colonization prevalence of various microbial species and isolates in the nasal samples collected from the cleaning staff. Gram-positive bacteria were predominant, constituting 92.7% of the samples, with coagulase-negative *Staphylococci* being the most prevalent (76.0%), notably *Staphylococcus epidermidis*. *Staphylococcus aureus* was detected in 22.9% of the samples, with 8.3% identified as methicillin-resistant. Among non-staphylococcal Gram-positive bacteria, *Micrococcus luteus* was the most common (8.3%). Gram-negative bacteria were present in 12.5% of the samples, primarily Enterobacteriaceae (12.5%), notably *Klebsiella* species (8.3%). On the other hand, Table 2b demonstrates the colonization prevalence of various microbial species and isolates in the hands samples and shows gram-positive bacteria dominance, 166 constituting 83.3% of the samples, with coagulase-negative *Staphylococci* being the most prevalent (74.0%). *Staphylococcus aureus* was detected in 6.3% of the samples, including 3.1% identified as MRSA. *Micrococcus luteus* was again prominent among non-staphylococcal Gram-positive bacteria (21.9%). Gram-negative bacteria were identified in 8.3% of the samples, primarily Enterobacteriaceae (4.2%), notably *Klebsiella* species (2.1%). Additionally, fungi (*Candida* species) were detected in 2.1% of hand samples.

### 3.4. Factors Associated with Colonization Prevalence

Due to the clinical significance of MRSA and Enterobacteriaceae in healthcare-associated infections, factors associated with their distribution were closely investigated. Results revealed in Table 3 show the association between the prevalence of MRSA and Enterobacteriaceae colonization in the nasal and hand samples of cleaning staff and various demographic and work-related factors. For nasal samples, there were no significant differences in colonization rates of both organisms across all variables except there was a significant association between the shift work and Enterobacteriaceae colonization (*p* = 0.028), with a higher prevalence observed among day-shift workers. Regarding hand samples no significant differences were observed in colonization rates for both organisms (*p* > 0.05) across different demographics, departments, methods of handwashing, and shifts. However, there was a significant association between the frequency of handwashing and MRSA colonization (*p* = 0.047), with the highest prevalence observed among those who washed their hands only after using the restroom (28.6%). Across different demographics, departments, frequency of handwashing and the method used, no significant differences were observed in the colonization rates for both organisms (*p* > 0.05) in combined (hands and nose) samples Table 3. However, a significant association was found between shift work and Enterobacteriaceae colonization (*p* = 0.008), with a higher prevalence among day-shift workers (23.3% vs. 2.8% positive).

### 3.5. Predictors Associated with MRSA and Enterobacteriaceae Colonization

In the multivariate binary logistic regression analyses for MRSA and Enterobacteriaceae colonization in the nasal samples of cleaning staff, various predictors were examined for their association with colonization. For MRSA, none of the predictors showed statistical significance Table 4a. However, for Enterobacteriaceae colonization, certain factors especially, age displayed a marginal association (*p* = 0.072), with a slight increase in odds (AOR = 1.150). Among nationalities, individuals from India had significantly lower odds of colonization (*p* = 0.043, AOR = 0.036). Night shift work was associated with significantly reduced odds of Enterobacteriaceae colonization (*p* = 0.016, AOR = 0.009). Notably, ICU and OR workers had substantially increased odds of colonization (*p* = 0.043, AOR = 177.838, *p* = 0.024, AOR = 126.055, respectively). Regarding hand hygiene practices, washing hands only after specific tasks was the reference category. Washing hands every hour was associated with significantly decreased odds of colonization (*p* = 0.023, AOR = 0.019). Additionally, using soap and water for handwashing was associated with lower odds of colonization (*p* = 0.043, AOR = 0.039) compared with alcohol-based hand hygiene Table 4b. In the multivariate binary logistic regression analyses examining predictors of MRSA, and Enterobacteriaceae colonization on the hands of cleaning staff, none of the factors emerged as statistically significant predictors for any of the isolated bacterial candidates. On the other hand, in multivariate binary logistic regression analyses assessing predictors of MRSA combined in both the nose and hands among cleaning staff, none of the factors demonstrated statistically significant associations Table 5a For Enterobacteriaceae 209 colonization, significant associations were observed with the night shift (AOR = 0.017, *p* = 0.015) and handwashing every hour (AOR = 0.024, *p* = 0.028) Table 5b. These findings suggest that cleaning staff working during the night shift have approximately 0.017 times the odds of Enterobacteriaceae colonization compared to those working during the day. Similarly, individuals who wash their hands every hour have approximately 0.024 times the odds of Enterobacteriaceae colonization compared to those who wash their hands after specific tasks.

## 4. Discussion

### 4.1. MRSA

In the current study, the possible role of cleaning staff in the transmission of HAIs’ contaminants among healthcare staff and patients enrolled in two primary healthcare facilities, in Eastern Province, Saudi Arabia, was investigated. The nose and hands of the cleaning staff were identified because they were recognized as the most significant reservoirs harboring healthcare-associated pathogens among workers and staff [21]. The hands are frequently implicated in the direct transmission of pathogens due to their constant contact with various surfaces, patients, and other staff members [22]. The nostrils, particularly the anterior nares, have been recognized as a primary colonization site for *Staphylococcus aureus*, including MRSA, which are significant contributors to HAIs [23]. The nose and hands of the cleaning staff were selected as sites of choice for microbiological identification, as they are regarded as the most significant reservoirs harboring healthcare-associated pathogens among workers and staff [25]. By targeting these sites, our study aims to capture a comprehensive snapshot of potential pathogen reservoirs among cleaning staff. The hands are frequently implicated in the direct transmission of pathogens due to their constant contact with various surfaces, patients, and other staff members [26]. The nostrils, particularly the anterior nares, have been recognized as a primary colonization site for *Staphylococcus aureus*, including MRSA, which are significant contributors to HAIs [27]. In our study, the colonization prevalence of MRSA was 3.1% in the hands and 8.3% in the nose. However, the prevalence was 9.4% in combined hand and nose samples. A systematic review by Dulan et al. in 2014 of 31 studies in Europe and the US to estimate the carriage of MRSA by healthcare workers, regardless of the sampling site, showed a pooled colonization prevalence of 1.8% [20]. Additionally, a systematic review and meta-analysis conducted by Horn W. et al., which included 42 studies on MRSA colonization of the hand among healthcare workers, reported a higher pooled colonization prevalence of 4.26%, compared to the 3.1% observed in our study [28]. Regarding nasal colonization among healthcare cleaning staff, studies in Canada, Brazil, Ecuador, and Uganda showed different nasal colonization rates for MRSA bacterial candidates of approximately 0%, 6%, 5%, and 13%, respectively [29,30,31,32]. Furthermore, MRSA colonization did not demonstrate any significant associations across the examined variables. Additionally, the study found that workers who only washed their hands after using the restroom exhibited higher rates of MRSA colonization, a finding consistent with the study conducted by Salmon et al. [33]. Therefore, ensuring frequent handwashing among cleaning staff is crucial to reducing the risk of HAI pathogen transmission.

### 4.2. Gram-Negative Pathogens

On the other hand, the prevalence of Gram-negative bacteria was 8.3% in the hands. Moreover, the prevalence of Enterobacteriaceae was found to be 12.5% in nasal samples and 4.2% in hand samples, while the overall 251 prevalence was estimated to be 15.6%. Notably, *Klebsiella* species emerged as the most dominant bacterial type across both anatomical sites. A comparative analysis involving a study by Larson et al. (1981) [34] with a cohort size of 106, reported a higher colonization prevalence of Gram-negative bacteria compared to our study (21–8.3%) with Enterobacteriaceae being the most identified Gram-negative pathogen. When comparing the prevalence of different Gram-negative pathogens in our study to the literature, we can observe significant discrepancies. For instance, a study by Ssemogerere et al. (2019) shows a high prevalence of *Pseudomonas* species, *Citrobacter* species, and *Acinetobacter*, while they found a low prevalence of *Klebsiella* species, in contrast to our study, where the most common Gram-negative pathogen was *klebsiella* species, while *Citrobacter* sp. and *Acinetobacter* sp. were not recorded [35]. Furthermore, our analysis revealed that an increased frequency of handwashing is associated with significantly lower odds of colonization with *Enterobacteriaceae*, particularly when handwashing occurs every hour. This observation is consistent with findings from a study conducted by Elaina, which highlighted similar outcomes regarding hand hygiene frequency. Moreover, like our study, no difference was reported between washing with water and soap and alcohol rub [36]. In contrast to the study conducted by Salmon S et al. in Vietnam, which states that alcohol-based rubs are more effective than soap and water in reducing bacterial colonization [33]. Although the prevalence of Enterobacteriaceae is highest in the outpatient department (28.6%), making it the most common location for such colonization, with the operating room (OR) being the second most common at 25%, our analysis indicates increased odds of Enterobacteriaceae colonization in the ICU and OR compared to other hospital departments. This observation is particularly significant given the increased vulnerability of patients in these settings to nosocomial infections. Additionally, lower odds of Enterobacteriaceae colonization were observed during the night shift, though the reasons for this remain unclear. Other potentially pathogenic microorganisms identified by a small subset of participants in our study included *Pseudomonas mendocina* and *Candida* spp. Concerning *Pseudomonas mendocina* documented cases of infection in immunocompetent individuals have been reported in various studies, as highlighted in a systematic review by Ioannou and Vougiouklakis [37]. In our study, colonization by *Pseudomonas mendocina* was observed in only two participants’ hands. Regarding *Candida* species, colonization was detected in two participants, one with *Candida albicans* and the other with *Candida tropicalis*. Both *Candida* species identified in our study can cause infections, from superficial to invasive candidiasis [38]. Sakita KM et al. conducted a study in an adult ICU, assessing the antifungal susceptibility of virulent *Candida* species. Isolated from healthcare workers’ hands and ICU surfaces revealed higher resistance rates among isolates from healthcare workers’ hands than those from ICU surfaces [39]. Since housekeepers have extensive contact with patient environments, they may inadvertently contribute to *Candida* transmission. Given that the study’s prevalence is low for *Pseudomonas mendocina* and *Candida* spp, their contribution to HAIs in the hospital remains unclear.

## 5. Strengths, Limitations, and Conclusions

This study provides novel insights into the potential role of hospital cleaning staff in harboring and potentially transmitting nosocomial pathogens. One of the key strengths of our study is that it is the first to specifically investigate the colonization of hospital cleaning staff with clinically significant nosocomial pathogens, addressing a previously overlooked population in infection control research. Unlike prior studies that focused primarily on healthcare professionals, this study highlights the potential contribution of hygiene staff to hospital contamination, emphasizing the importance of infection prevention strategies targeting this group.

Another strength of this study is its structured approach, which included both nasal and hand sampling to provide a more comprehensive assessment of colonization patterns. By evaluating two distinct anatomical sites, we were able to gain deeper insight into potential reservoirs of transmission within this workforce. Additionally, the inclusion of multiple hospital departments and shifts strengthens the generalizability of our findings across different healthcare settings.

Despite its strengths, our study has several limitations. A key limitation is the absence of patient or environmental sampling, which prevents us from directly confirming transmission pathways between cleaning staff, patients, and hospital surfaces. Although we demonstrated colonization, we did not assess whether these bacteria were actively transmitted to other individuals or inanimate objects within the hospital setting. Future studies incorporating environmental sampling and patient monitoring are necessary to establish direct transmission routes. Another limitation is that colonization does not necessarily equate to infection, and additional methods could be utilized in future research to distinguish between transient and persistent colonization.

Regarding the collection of nasal samples, we recognize the concern about whether nasal colonization is relevant when studying MRSA and Enterobacteriaceae, given that these pathogens are primarily transmitted via direct contact. However, nasal colonization is well-documented as a potential reservoir for MRSA, which can subsequently be transferred to the hands and other surfaces. This is particularly relevant in individuals who frequently touch their faces during routine activities. Therefore, we included nasal sampling in our study to assess colonization reservoirs that could contribute to potential hand contamination, thereby reinforcing the rationale for studying hygiene staff as potential vectors.

We also acknowledge the absence of resistance profiling for Gram-negative bacteria as a limitation. While this would provide additional insights, our primary objective was to assess whether cleaning staff serve as reservoirs of nosocomial pathogens—a previously unexamined question. Future studies should incorporate resistance profiling to better understand antimicrobial resistance patterns in this population.

Additionally, the presence of these pathogens in cleaning staff also implies an occupational health risk, as they themselves may be at increased risk of healthcare-associated infections. This highlights the need for enhanced infection control protocols not only to mitigate potential pathogen transmission to patients but also to safeguard cleaning staff from exposure to antimicrobial-resistant organisms.

In conclusion, although we did not establish direct evidence of transmission, our findings suggest that cleaning staff, similar to healthcare professionals, may play a role in the persistence of nosocomial pathogens in healthcare settings. To mitigate these risks, infection control strategies should emphasize regular hand hygiene training, enhanced decolonization strategies, and targeted educational programs for hospital hygiene staff. By prioritizing infection prevention measures among this often-overlooked workforce, hospitals can further strengthen their overall strategies for reducing healthcare-associated infections.

## Figures and Tables

**Table 1:** Sociodemographic characteristics and handwashing practices of the participants.

			M	SD	N	%
Age			42.2	8.7		
Age (Binned)	21–30				11	11.5%
	31–40				29	30.2%
	41–50				40	41.7%
	51–60				15	15.6%
	>60				1	1.0%
Gender	Male				74	77.1%
	Female				22	22.9%
Nationality	Bangladesh				40	41.7%
	Philippines				21	21.9%
	India				20	20.8%
	Nepal				14	14.6%
	Sri Lanka				1	1.0%
Shift	Day				60	62.5%
	Night				36	37.5%
Department	Inpatient				72	75.0%
		Wards			30	31.3%
		Emergency room			16	16.7%
		ICU			10	10.4%
		Psychiatry ward			8	8.3%
		OR			8	8.3%
	Outpatient				24	25.0%
		Outpatient clinics			14	14.6%
		Family medicine center			10	10.4%
Frequency of handwashing		After specific tasks			67	69.8%
		Every hour			19	19.8%
		Only after using the restroom			7	7.3%
		Only when hands are visibly dirty			3	3.1%
Methods used for hand cleaning		Soap and water			82	85.4%
		Alcohol			14	14.6%
Total					96	100.0%

“Psychiatry ward” is located in a separate building. M: mean, SD: standard deviation, N: number.

**Table 2:** (**a**) Colonization prevalence of each bacterial candidate isolated from nose and hands of the cleaning staff. (**b**) Colonization prevalence of each bacterial candidate isolated from the hands of the cleaning staff.

**(a)**					
				**N**	**% (N = 96)**
N/G				5	5.2%
**Gram Positive**				89	92.7%
	Coagulase-Negative *Staphylococci*			73	76.0%
		*Staphylococcus epidermidis*		55	57.3%
		*Staphylococcus capitis*		9	9.4%
		*Staphylococcus warneri*		7	7.3%
		*Staphylococcus lugdunensis*		5	5.2%
		*staphylococcus haemolyticus*		4	4.2%
		*Staphylococcus cohnii* spp.		2	2.1%
		*Staphylococcus hominis*		1	1.0%
	*Staphylococcus aureus*			22	22.9%
		MRSA (Methicillin-resistant *Staphlycoccus auerus*)		8	8.3%
		MSSA		14	14.6%
	Non-Staph Gram Positive			16	16.7%
		*Micrococcus luteus*		8	8.3%
		*Corynebacterium species*		6	6.3%
			*Corynebacterium pseudodiphtheriticum*	3	3.1%
			*Corynebacterium propinquum*	2	2.1%
			*Corynebacterium accolens*	1	1.0%
		*Bacillus lechmiformis*		1	1.0%
		*Streptococcus mitis*		1	1.0%
**Gram Negative**				12	12.5%
	*Enterobacteriaceae*			12	12.5%
		*Klebsiella species*		8	8.3%
			*Klebsiella aerogenes*	7	7.3%
			*Klebsiella oxytoca*	1	1.0%
		*Citrobacter koseri*		1	1.0%
		*Citrobacteer fereeundii*		1	1.0%
		*Proteus mirablis*		1	1.0%
		*Escherichia coli*		1	1.0%
	Non-*Enterobacteriaceae*			1	1.0%
		*Pseudomonas leuteola*		1	1.0%
**(b)**					
				**N**	**% (N = 96)**
N/G				13	13.5%
**Gram Positive**				80	83.3%
	CNS (Coagulase Negative *Staphylococci*)			71	74.0%
		*Staphylococcus epidermidis*		38	39.6%
		*Staphylococcus capitis*		17	17.7%
		*Staphylococcus warneri*		19	19.8%
		*Staphylococcus haemolyticus*		12	12.5%
		*Staphylococcus hominis*		11	11.5%
		*Staphylococcus pasteuri*		5	5.2%
		*Staphylococcus saprophyticus*		2	2.1%
		*Staphylococcus arlttae*		2	2.1%
		*Staphylococcus cohnii* spp.		1	1.0%
		*Staphylococcus auricularis*		1	1.0%
	*Staphylococcus aureus*			6	6.3%
		MRSA		3	3.1%
		MSSA		3	3.1%
	Non- Staph Gram Positive			25	26.0%
		*Micrococcus luteus*		21	21.9%
		*Kocuria rhizophila*		2	2.1%
		*Rothia kristinae*		1	1.0%
		*Brevibacterium casei*		1	1.0%
**Gram Negative**				8	8.3%
	*Enterobacteriaceae*			4	4.2%
		*Klebsiella species*		2	2.1%
			*Klebsiella aerogenes*	1	1.0%
			*Klebsiella pneumonia*	1	1.0%
		*Enterobacter*		1	1.0%
			*Enterobacter hormaechei*	1	1.0%
			*Enterobacter asburiae*	1	1.0%
			*Enterobaacter cloace*	1	1.0%
		*Escherichia coli*		1	1.0%
	Non-*Enterobacteriaceae*			4	4.2%
		*Pseudomanas mendocina/olevorans*		2	2.1%
		*Neisseria flava/perflava/subflava*		1	1.0%
		*Ochrobactrum intermedium (Brucella intermedia)*		1	1.0%
**Fungi (*Candida* Species)**				2	2.1%
		*Candida albicans*		1	1.0%
		*Candida tropicalis*		1	1.0%

Note: (**a**) The prevalence of each isolate may not add up to be equal to the prevalence of that genus because some participants had more than isolate (of the same and/or different genus). (**b**) N: number, N/G: no growth.

**Table 3:** Association of colonization prevalence of MRSA and Enterobacteriaceae in nose and Hands with demographics, department, shift, frequency and method of handwashing.

		MRSA Positive Isolates						Enterobacteriaceae Isolates					
		Hands		Nose		Total		Hands		Nose		Total	
		*N* (%)	*p*	*N* (%)	*p*	*N* (%)	*p*	*N* (%)	*p*	*N* (%)	*p*	*N* (%)	*p*
Age	21–30	0 (0.0)	0.842	0 (0.0)	0.777	0 (0.0)	0.660	0 (0.0)	0.898	0 (0.0)	0.358	0 (0.0)	0.474
	31–40	1 (3.4)		3 (10.3)		4 (13.8)		2 (6.9)		2 (6.9)		4 (13.8)	
	41–50	1 (2.5)		3 (7.5)		3 (7.5)		2 (5.0)		8 (20.0)		9 (22.5)	
	51–60	1 (6.7)		2 (13.3)		2 (13.3)		0 (0.0)		2 (13.3)		2 (13.3)	
	>60	0 (0.0)		0 (0.0)		0 (0.0)		0 (0.0)		0 (0.0)		0 (0.0)	
Gender	Female	1 (4.5)	0.546	2 (9.1)	1.000	2 (9.1)	1.000	2 (9.1)	0.224	1 (4.5)	0.285	3 (13.6)	1.000
	Male	2 (2.7)		6 (8.1)		7 (9.5)		2 (2.7)		11 (14.9)		12 (16.2)	
Nationality	Bangladesh	0 (0.0)	0.240	2 (5.0)	0.688	2 (5.0)	0.397	2 (5.0)	0.523	8 (20.0)	0.469	9 (22.5)	0.624
	India	1 (5.0)		2 (10.0)		2 (10.0)		0 (0.0)		2 (10.0)		2 (10.0)	
	Nepal	1 (7.1)		2 (14.3)		3 (21.4)		0 (0.0)		1 (7.1)		1 (7.1)	
	Philippines	1 (4.8)		2 (9.5)		2 (9.5)		2 (9.5)		1 (4.8)		3 (14.3)	
	Sri Lanka	0 (0.0)		0 (0.0)		0 (0.0)		0 (0.0)		0 (0.0)		0 (0.0)	
Shift	Day	1 (1.7)	0.559	4 (6.7)	0.468	4 (6.7)	0.289	4 (6.7)	0.523	11 (18.3)	0.028 *	14 (23.3)	0.008 *
	Night	2 (5.6)		4 (11.1)		5 (13.9)		0 (0.0)		1 (2.8)		1 (2.8)	
Department	Emergency Room	0 (0.0)	0.904	1 (6.3)	0.785	1 (6.3)	0.757	1 (6.3)	0.888	1 (6.3)	0.312	2 (12.5)	0.417
	Family Medicine Center	0 (0.0)		0 (0.0)		0 (0.0)		0 (0.0)		0 (0.0)		0 (0.0)	
	ICU	0 (0.0)		2 (20.0)		2 (20.0)		0 (0.0)		1 (10.0)		1 (10.0)	
	OR	0 (0.0)		1 (12.5)		1 (12.5)		0 (0.0)		2 (25.0)		2 (25.0)	
	Outpatient Clinics	1 (7.1)		1 (7.1)		2 (14.3)		0 (0.0)		4 (28.6)		4 (28.6)	
	Psychiatry Ward	0 (0.0)		0 (0.0)		0 (0.0)		0 (0.0)		0 (0.0)		0 (0.0)	
	Wards	2 (6.7)		3 (10.0)		3 (10.0)		3 (10.0)		4 (13.3)		6 (20.0)	
Department (Overall)	Inpatient	2 (2.8)	1.000	7 (9.7)	0.675	7 (9.7)	1.000	4 (5.6)	0.569	8 (11.1)	0.487	11 (15.3)	1.000
	Outpatient	1 (4.2)		1 (4.2)		2 (8.3)		0 (0.0)		4 (16.7)		4 (16.7)	
Frequency of Handwashing	After Specific Tasks	1 (1.5)	0.047 *	5 (7.5)	0.647	5 (7.5)	0.247	4 (6.0)	0.726	10 (14.9)	0.291	13 (19.4)	0.189
	Every Hour	0 (0.0)		2 (10.5)		2 (10.5)		0 (0.0)		1 (5.3)		1 (5.3)	
	Only after Using the Restroom	2 (28.6)		1 (14.3)		2 (28.6)		0 (0.0)		0 (0.0)		0 (0.0)	
	Only when Hands Are Visibly Dirty	0 (0.0)		0 (0.0)		0 (0.0)		0 (0.0)		1 (33.3)		1 (33.3)	
Method of Handwashing	Alcohol	0 (0.0)	1.000	1 (7.1)	1.000	1 (7.1)	1.000	0 (0.0)	1.000	2 (14.3)	0.686	2 (14.3)	1.000
	Soap and Water	3 (3.7)		7 (8.5)		8 (9.8)		4 (4.9)		10 (12.2)		13 (15.9)	

* Is used for statistically significant *p* values (less than 0.05).

**Table 4:** (**a**) Multivariate binary logistic regression analysis of predictors of colonization of MRSA in the nose and hands of participants. (**b**) Multivariate binary logistic regression analysis of predictors of colonization of Enterobacteriaceae in nose and hands of participants.

**(a)**													
**Nose**			**B**	**Adjusted Odds Ratio (AOR)**	**95% C.I. for AOR**		***p* Value**	**Hands**	**B**	**Adjusted Odds Ratio (AOR)**	**95% C.I. for AOR**		***p* Value**
					**Lower**	**Upper**					**Lower**	**Upper**	
MRSA	Age (years)		0.017	1.017	0.918	1.127	0.744		0.079	1.082	0.903	1.295	0.393
	Gender	Female	Ref	Ref	Ref	Ref	Ref		Ref	Ref	Ref	Ref	Ref
		Male	0.034	1.035	0.003	334.475	0.991		−59.557	0	0	.	1
	Nationality						0.805						1
		Bangladesh	Ref	Ref	Ref	Ref	Ref		Ref	Ref	Ref	Ref	Ref
		India	0.115	1.122	0.116	10.814	0.921		31.208	3.58 × 10^13^	0	.	0.996
		Nepal	1.965	7.132	0.319	159.321	0.215		34.873	1.4 × 10^15^	0	.	0.998
		Philippines	0.261	1.298	0.004	428.614	0.93		3.769	43.316	0	.	1
		Sri Lanka	0.739	2.094	0	.	1		−13.615	0	0	.	1
	Shift	Day	Ref	Ref	Ref	Ref	Ref		Ref	Ref	Ref	Ref	Ref
		Night	0.094	1.099	0.189	6.4	0.917		−14.67	0	0	.	0.996
	Department						0.942						1
		Emergency	Ref	Ref	Ref	Ref	Ref		Ref	Ref	Ref	Ref	Ref
		Family Medicine	−18.874	0	0	.	0.999		15.079	3,537,064	0	.	0.999
		ICU	1.462	4.314	0.226	82.264	0.331		13.723	911,758	0	.	0.999
		OR	−0.057	0.945	0.022	40.497	0.976		41.753	1.36 × 10^18^	0	.	0.998
		Outpatient	−0.725	0.484	0.011	21.086	0.706		29.858	9.27 × 10^12^	0	.	0.998
		Psychiatry Ward	−19.673	0	0	.	0.999		9.839	18,749.52	0	.	1
		Wards	0.221	1.247	0.092	16.931	0.868		15.367	4,716,658	0	.	0.998
	Frequency of handwashing						0.919						1
		After specific tasks	Ref	Ref	Ref	Ref	Ref		Ref	Ref	Ref	Ref	Ref
		Every hour	−0.494	0.61	0.065	5.757	0.666		−15.798	0	0	.	0.998
		Only after using the restroom	0.817	2.264	0.142	36.007	0.563		48.63	1.32 × 10^21^	0	.	0.994
		Only when hands are visibly dirty	−17.633	0	0	.	0.999		30.936	2.73 × 10^13^	0	.	0.999
	Method of handwashing	Alcohol	Ref	Ref	Ref	Ref	Ref		Ref	Ref	Ref	Ref	Ref
		Soap and Water	0.426	1.531	0.066	35.417	0.791		16.235	11,243,847	0	.	0.999
	Constant		−3.973	0.019			0.356		−41.101	0			1
**(b)**													
**Nose**			**B**	**Adjusted Odds Ratio (AOR)**	**95% C.I. for AOR**		***p* Value**	**Hands**	**B**	**Adjusted Odds Ratio (AOR)**	**95% C.I. for AOR**		***p* Value**
					**Lower**	**Upper**					**Lower**	**Upper**	
*Enterobacteriaceae*	Age (years)		0.14	1.15	0.988	1.34	0.072		−0.055	0.946	0.813	1.102	0.477
	Gender	Female	Ref	Ref	Ref	Ref	Ref		Ref	Ref	Ref	Ref	Ref
		Male	−1.14	0.32	0	33,241.26	0.847		0.524	1.688	0	.	1
	Nationality						0.329						1
		Bangladesh	Ref	Ref	Ref	Ref	Ref		Ref	Ref	Ref	Ref	Ref
		India	−3.312	0.036	0.001	0.906	0.043 *		−17.376	0	0	.	0.998
		Nepal	−2.577	0.076	0.003	2.251	0.136		1.374	3.95	0	.	1
		Philippines	−3.933	0.02	0	2172.737	0.507		0.925	2.522	0	.	1
		Sri Lanka	0.71	2.034	0	.	1		−0.083	0.92	0	.	1
	Shift	Day	Ref	Ref	Ref	Ref	Ref		Ref	Ref	Ref	Ref	Ref
		Night	−4.711	0.009	0	0.416	0.016 *		−16.707	0	0	.	0.998
	Department						0.355						1
		Emergency	Ref	Ref	Ref	Ref	Ref		Ref	Ref	Ref	Ref	Ref
		Family Medicine	−19.122	0	0	.	0.999		−20.111	0	0	.	0.999
		ICU	5.181	177.838	1.165	27,146	0.043 *		−1.767	0.171	0	.	1
		OR	4.837	126.055	1.912	8308.754	0.024 *		−17.859	0	0	.	0.999
		Outpatient	3.389	29.641	0.551	1594.342	0.096		−19.422	0	0	.	0.999
		Psychiatry Ward	−16.847	0	0	.	0.999		−17.718	0	0	.	0.999
		Wards	0.801	2.229	0.118	42.203	0.593		−0.364	0.695	0.046	10.479	0.793
	Frequency of handwashing						0.158						1
		After specific tasks	Ref	Ref	Ref	Ref	Ref		Ref	Ref	Ref	Ref	Ref
		Every hour	−3.963	0.019	0.001	0.584	0.023 *		−18.232	0	0	.	0.998
		Only after using the restroom	−17.745	0	0	.	0.999		−17.113	0	0	.	0.999
		Only when hands are visibly dirty	2.351	10.494	0	41,524,934	0.762		−2.298	0.1	0	.	1
	Method of handwashing	Alcohol	Ref	Ref	Ref	Ref	Ref		Ref	Ref	Ref	Ref	Ref
		Soap and Water	−3.25	0.039	0.002	0.903	0.043 *		18.464	1.04 × 10^8^	0	.	0.999
	Constant		−3.464	0.031			0.612		−17.928	0			0.999

* *p* < 0.05, Significant.

**Table 5:** (**a**) Multivariate binary logistic regression analysis of predictors of colonization of MRSA in nose and hands of participants. (**b**) Multivariate binary logistic regression analysis of predictors of colonization of Enterobacteriaceae in nose and hand of participants.

(**a**)							
			**B**	**Adjusted Odds Ratio (AOR)**	**95% C.I. for AOR**		***p* Value**
					**Lower**	**Upper**	
MRSA	Age (years)		−0.006	0.994	0.892	1.107	0.910
	Gender	Female	Ref	Ref	Ref	Ref	Ref
		Male	−0.505	0.604	0.003	126.929	0.853
	Nationality						0.564
		Bangladesh	Ref	Ref	Ref	Ref	Ref
		India	0.019	1.019	0.101	10.268	0.987
		Nepal	2.538	12.648	0.565	283.176	0.110
		Philippines	−0.100	0.905	0.004	194.735	0.971
		Sri Lanka	0.584	1.794	0.000	.	1.000
	Shift	Day	Ref	Ref	Ref	Ref	Ref
		Night	0.173	1.189	0.200	7.058	0.849
	Department						0.930
		Emergency	Ref	Ref	Ref	Ref	Ref
		Family Medicine	−19.417	0.000	0.000	.	0.999
		ICU	1.624	5.072	0.235	109.583	0.300
		OR	−0.307	0.736	0.014	38.656	0.879
		Outpatient	−0.146	0.864	0.022	34.039	0.938
		Psychiatry Ward	−20.478	0.000	0.000	.	0.999
		Wards	0.116	1.123	0.077	16.443	0.933
	Frequency of handwashing						0.530
		After specific tasks	Ref	Ref	Ref	Ref	Ref
		Every hour	−0.588	0.556	0.058	5.301	0.610
		Only after using the restroom	1.896	6.658	0.484	91.564	0.156
		Only when hands are visibly dirty	−17.717	0.000	0.000	.	0.999
	Method of handwashing	Alcohol	Ref	Ref	Ref	Ref	Ref
		Soap and Water	0.460	1.584	0.064	38.998	0.778
	Constant		−2.656	0.070			0.532
(**b**)							
			**B**	**Adjusted Odds Ratio (AOR)**	**95% C.I. for AOR**		***p* Value**
					**Lower**	**Upper**	
*Enterobacteriaceae*	Age (years)		0.054	1.055	0.954	1.167	0.298
	Gender	Female	Ref	Ref	Ref	Ref	Ref
		Male	−1.947	0.143	0.001	32.528	0.482
	Nationality						0.377
		Bangladesh	Ref	Ref	Ref	Ref	Ref
		India	−2.390	0.092	0.007	1.134	0.063
		Nepal	−1.883	0.152	0.008	2.894	0.210
		Philippines	−2.732	0.065	0.000	14.304	0.321
		Sri Lanka	−0.691	0.501	0.000	.	1.000
	Shift	Day	Ref	Ref	Ref	Ref	Ref
		Night	−4.047	0.017	0.001	0.451	0.015 *
	Department						0.570
		Emergency	Ref	Ref	Ref	Ref	Ref
		Family Medicine	−20.982	0.000	0.000	.	0.999
		ICU	3.057	21.267	0.452	1000.998	0.120
		OR	3.827	45.912	0.913	2307.729	0.056
		Outpatient	1.773	5.889	0.336	103.103	0.225
		Psychiatry Ward	−17.783	0.000	0.000	.	0.999
		Wards	0.129	1.138	0.132	9.789	0.906
	Frequency of handwashing						0.175
		After specific tasks	Ref	Ref	Ref	Ref	Ref
		Every hour	−3.726	0.024	0.001	0.671	0.028 *
		Only after using the restroom	−18.303	0.000	0.000	.	0.999
		Only when hands are visibly dirty	1.754	5.780	0.004	8541.590	0.638
	Method of handwashing	Alcohol	Ref	Ref	Ref	Ref	Ref
		Soap and Water	−1.586	0.205	0.018	2.330	0.201
	Constant		0.552	1.736			0.885

* *p* < 0.05, Significant.

## Data Availability

The data used in this study are not publicly available due to privacy concerns and institutional regulations. However, de-identified data supporting the findings of this study may be available from the corresponding author upon reasonable request, provided it complies with institutional data-sharing policies and privacy requirements.
